# Compaction-Aware Flash Memory Remapping for Key–Value Stores †

**DOI:** 10.3390/mi16060699

**Published:** 2025-06-11

**Authors:** Jialin Wang, Zhen Yang, Yi Fan, Yajuan Du

**Affiliations:** 1 College of Electrical Engineering, Naval University of Engineering, Wuhan 430033, China; 1310041031@nue.edu.cn; 2 School of Computer Science and Artificial Intelligence, Wuhan University of Technology, Wuhan 430070, China; zhenyoung@whut.edu.cn (Z.Y.); fy2685018622@whut.edu.cn (Y.F.)

**Keywords:** LSM-tree, memory, compaction, SSD, data remapping

## Abstract

With the rapid development of big data and artificial intelligence, the demand for memory has exploded. As a key data structure in modern databases and distributed storage systems, the Log-Structured Merge Tree (LSM-tree) has been widely employed (such as LevelDB, RocksDB, etc.) in systems based on key–value pairs due to its efficient writing performance. In LSM-tree-based KV stores, typically deployed on systems with DRAM-SSD storage, the KV items are first organized into MemTable as buffer for SSTables in main memory. When the buffer size exceeds the threshold, MemTable is flushed to the SSD and reorganized into an SSTable, which is then passed down level by level through compaction. However, the compaction degrades write performance and SSD endurance due to significant write amplification. To address this issue, recent proposals have mostly focused on redesigning the structure of LSM trees. We discover the prevalence of unchanged data blocks (UDBs) in the LSM-tree compaction process, i.e., UDBs are written back to SSD the same as they are read into memory, which induces extra write amplification and degrades I/O performance. In this paper, we propose a KV store design in SSD, called RemapCom, to exploit remapping on these UDBs. RemapCom first identifies UDBs with a lightweight state machine integrated into the compaction merge process. In order to increase the ratio of UDBs, RemapCom further designs a UDB retention method to further develop the benefit of remapping. Moreover, we implement a prototype of RemapCom on LevelDB by providing two primitives for the remapping. Compared to the state of the art, the evaluation results demonstrate that RemapCom can reduce write amplification by up to 53% and improve write throughput by up to 30%.

## 1. Introduction

With the development of Big Data, the need for efficient and scalable memory systems has become increasingly critical. There are more and more new types of memory being designed and manufactured. Optimization methods on memories like resistive RAM (RRAM) [1], magnetoresistive RAM (MRAM) [2], phase-change memory (PCM) [3], and flash memory are being studied more. The devices based on flash memory are especially popular in the consumer market. Compared with the flash memory, low maturity of process manufacturing like high integration difficulty of other memories like MRAM and RRAM leads to high manufacturing costs and great challenges in yield management, making it difficult to mass-produce like flash memory. Moreover, the high power consumption also makes it unsuitable for mobile devices such as smartphones and laptops, and the recently proposed 3D stacking technologies have made a qualitative leap in the storage density of NAND flash memory. As a result, flash memory has become the optimal solution for persistent storage in terms of comprehensive cost, access speed, and power consumption. Nowadays, the flash-based devices like SD card and SSD are applied to various electronic products.

The structure of the Log-Structured Merge (LSM) Tree [4,5,6] has been widely used in key–value (KV)-based databases such as LevelDB [7], RocksDB [8], HBase [9], and TiDB [10]. By directly appending data, random KV writes can be converted into sequential ones, resulting in improved write performance. KV data are first organized to be data blocks, and then multiple data blocks are formed in a Sorted String Table (SSTable). SSTables are stored in multiple levels on the disks. When storage volume increases, compaction is triggered to merge new data and old data [11]. In detail, SSTables in the higher level are first loaded into memory, together with the overlapping SSTables in the lower level. Then, the KV items in these SSTables are sorted to merge data with the same keys. Subsequently, the valid KV data are reorganized to be new SSTables and written back to disk. In this process, these valid data are loaded and written back, which induces I/O overhead and duplicate writes.

In order to satisfy the requirement of fast data storage, replacement of HDDs with Solid-State Drives (SSDs) in KV storage systems has been considered [12,13]. However, it is still challenging to realize an efficient SSD-based KV store due to periodic SSTable compaction. As SSDs update data out of place, the old data are not deleted immediately. Studies are in place to deal with duplicate writes during data copy or journaling [14,15,16,17]. These studies propose SSD remap strategies to transfer the overhead of physical data migration to mapping of the new logical address to the old physical address. These techniques allow the old pages to be reused, thus alleviating write amplification within the SSDs.

In this paper, we address the duplicate write issue in SSDs due to compaction, a core operation of LSM-tree-based KV stores. Specifically, we consider applying the remapping strategy to alleviate the compaction overhead on I/O performance and write amplification. There are two challenges in realizing the remapping in the compaction of LSM trees. First, we need to consider the granularity of duplicate data. As remapping can only be implemented in flash page granularity, it is critical to determine the granularity of the duplicate data during compaction. Second, there needs to be a way to figure out the duplicate writes. That is, there are no explicit data copy operations involved in moving duplicate pages during the compaction process. Thus, an efficient means of identifying duplicate writes needs to be devised.

In order to optimize compaction performance in LSM-tree-based KV store, this paper proposes a remapping-based compaction method, which we call RemapCom. First, we notice that the size of a data block (typically 4 KB) is often similar to the size of an SSD page. Thus, we determine the data block to be the remapping granularity. Second, we consider the identification and retention of duplicate data during compaction, which we refer to as unchanged data blocks (UDBs). Our preliminary analysis, which we discuss later, shows that there exists a large portion of UDB in real-world benchmarks. Then, in order to identify UDB, we design a lightweight state machine to track the status of the KV items in each data block, with the assistance of a lazy write-back scheme. Subsequently, to take full advantage of the remapping benefit, we design a UDB retention strategy to prevent data blocks from being separated due to adjacent intersecting blocks. This improves the ratio of UDB and further optimizes system performance. Finally, we implement two primitives in RemapCom to remap the new logical address onto the old physical address of these UDBs to avoid duplicate writes.

We implement RemapCom in LevelDB and perform experimental evaluations with db_bench and real-world benchmarks which are YCSB [18] and Mixgraph [19]. Results show that a large portion of data blocks remain unchanged during compaction and that RemapCom can significantly reduce write amplification and improve LevelDB write performance.

The contributions of this paper are summarized as follows.

- We perform a preliminary study to discover the prevalence of UDB in real-world benchmarks.
- We propose RemapCom, an SSD remapping-based compaction method for LSM-tree-based KV stores. In RemapCom, we design a lightweight state machine to identify duplicate data caused by the compaction process.
- We design a UDB retention strategy in RemapCom to increase the ratio of UDB, in order to better exploit the benefit of UDB remapping.
- We implement RemapCom in LevelDB by providing two primitives, getLPN and remap, to support data block remapping.
- We evaluate RemapCom in real-world benchmarks and experimental results show that it can reduce the write amplification by up to 53% and improve the write throughput by up to 30%.

The remainder of the paper is organized as follows. Section 2 introduces the background of SSDs and LSM trees. Section 3 illustrates our preliminary study and the motivation of RemapCom. Section 4 presents the detailed design of RemapCom. Section 5 demonstrates the setup and results of the experiment. Section 6 introduces related work, and Section 7 concludes this paper.

## 2. Background

In this section, we first present the background of flash-based SSDs. Next, existing work on SSD remap strategy is illustrated. Finally, we present the basics of LSM-trees.

### 2.1. Flash-Based SSD

In flash-based SSDs, there are two core components: the SSD controller and flash chips. The function of the SSD controller is to handle read and write operations and manage the flash chips via logical-to-physical (L2P) address mapping, garbage collection (GC), and other functions. Flash chips are organized hierarchically, from small to large, into pages, blocks, planes, and chips [20]. Page is the unit to perform read and write, while block is the unit to perform data erasures [21,22,23,24].

Unlike hard drives, where data can be directly overwritten, a flash-based SSD requires a block to be erased before it can be written again. This makes in-place updates inefficient; thus, SSDs use out-of-place updates [25]. More specifically, when data need to be updated, instead of overwriting the existing data, the SSD writes the new data to a new location on the flash memory. Then, the SSD controller updates the L2P mapping table in the Flash Translation Layer (FTL) to have the logical page number (LPN) point to the new physical page number (PPN). Then, the old physical page that contains the previous version of the data is marked as invalid.

SSDs utilize GC to erase blocks, enabling the rewriting of pages within these blocks. Each flash page is equipped with an Out-of-Band (OOB) space to store information about the corresponding logical page, forming a reverse L2P mapping table, known as the Physical-to-Logical (P2L) table. During the process of GC, a victim block is first chosen, and then the valid pages within that block have to be migrated to another block. Subsequently, FTL establishes the new mappings in the P2L and L2P tables. Finally, the block is erased.

### 2.2. Remapping-Based SSDs

**Figure 1:**
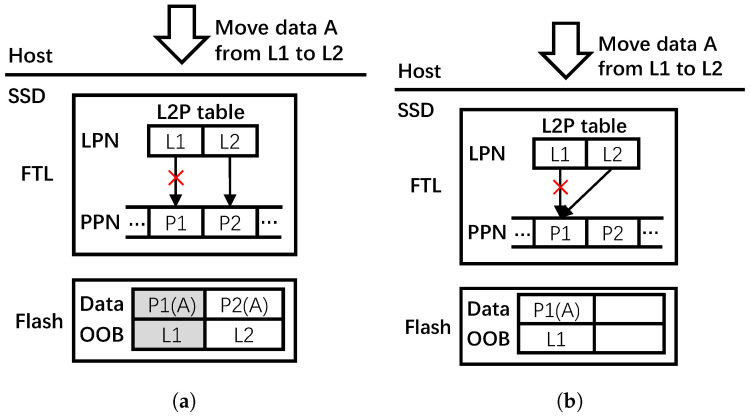
Writes in SSDs for data moving by host. (**a**) Conventional approach. (**b**) Remapping approach.

SSD remap strategy is a way to reuse the duplicate data involved in data copying, data moving, and journaling by just modifying the mappings in FTL [26,27,28,29]. When the host moves data from old pages to new pages, the conventional approach would first have the data copied into new pages and have the FTL establish the new mappings, then mark the old pages invalid and delete the old L2P mappings. For example, in Figure 1a, suppose that the host is to move data A from logical page L1 to L2, the conventional approach first locates the physical page P1 through the L2P table. Then, the data of page P1 is copied to a new physical page P2. Subsequently, a new mapping of L2→P2 is established in the L2P table, after which P1 is marked invalid and the mapping of L1→P1 is eliminated. The new P2L mapping is written into the OOB space of page P2. Note that P1 can be written again only after GC.

In the remapping approach, changing the old L2P mapping to the mapping between the new logical address and the old physical address is all that is needed, and the old data still remain valid. Figure 1b shows an example of the remapping approach. Observe that the FTL just needs to establish a new mapping of L2→P1 and then eliminate the old mapping of L1→P1. Compared with the conventional approach, the remapping approach reduces write amplification, thereby improving write performance and endurance of the SSD.

Mapping consistency is an issue that needs to be considered in the remapping approach [26,27,28,30]. Due to the out-of-place update characteristics of flash memory, after remapping, the relevant P2L mappings in the OOB space of flash pages cannot be modified even as the L2P mappings have changed, as shown in Figure 1b. This becomes a problem as a wrong L2P table may be rebuilt during GC and power-off recovery. Existing works propose to maintain a remap table, which holds the remapping relationships, to solve this mapping consistency problem [26,27,28]. During GC, FTL first scans the remap table, and the FTL will use these entries to build its L2P table if entries belonging to any physical pages are found in the victim block. Then, these entries are cleared in the remap table. In the case of combining mapping table with original P2L mappings, the FTL can rebuild the L2P table correctly.

### 2.3. Log-Structured Merge Trees

**Figure 2:**
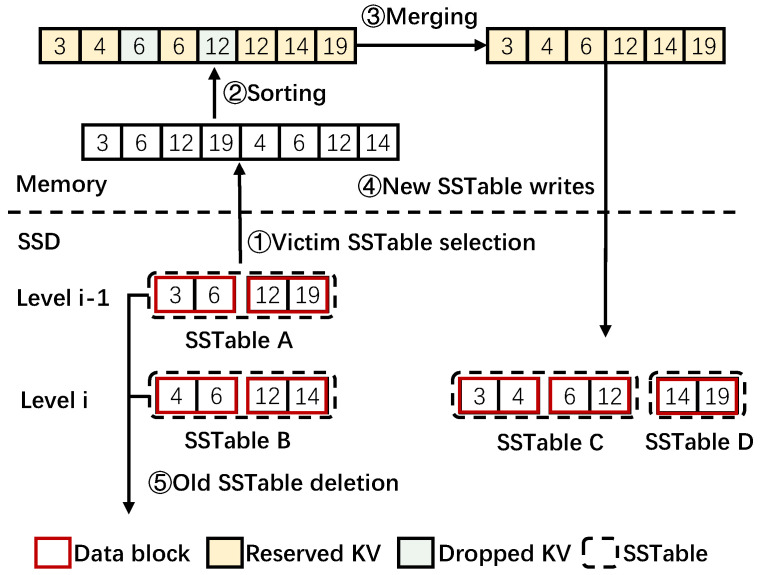
Compaction process of LSM-tree.

Log-Structured Merge Trees organize data to be a memtable in memory and SSTable in SSDs [5,31]. When the host sends a put operation, KV items are initially inserted into the memtable. When the memtable reaches a pre-determined size, it transforms into an immutable memtable, which cannot receive new KV items, and it is flushed to organize an SSTable. In SSDs, SSTables are organized in multiple levels. KV items in an SSTable are organized into multiple data blocks, each of which has a certain size (often 4 KB by default) and is sorted in order of keys. For example, in Figure 2, a SSTable in Level i−1 contains two data blocks. One contains keys of 3 and 6, while the other contains keys of 12 and 19. As the storage volume increases, an SSTable in a higher level would be compacted into a lower level by the compaction process. The compaction process can be summarized into five steps, as shown in the example of Figure 2.

*Step* ➀*: Victim SSTable selection.* Select a SSTable in the level of $L_{i-1}$ as the victim and find SSTables in the next lower level $L_{i}$ whose key ranges overlap with the victim SSTable. Note that $L_{0}$ is an exception since SSTables in $L_{0}$ may overlap with each other. Therefore, several overlapping SSTables in $L_{0}$ and SSTables with overlapping keys in $L_{1}$ would be selected. The SSTables from $L_{i}$ and $L_{i-1}$ are all read into memory, as shown in Figure 2.

*Step* ➁*: KV sorting.* The KV items in the victim SSTable A and overlapped SSTable B are combined together and sorted in ascending order of keys.

*Step* ➂*: KV merging.* The sorted KV items are merged. In detail, compaction traverses the sorted KV items one by one. The KV items are dropped if they are denoted as invalid or old versions of the same keys. For example, the KVs of 6 and 12 in Figure 2 are dropped. Otherwise, the KV items would be reserved. Finally, the reserved KV items are reorganized as new SSTables.

*Step* ➃*: New SSTable writes.* The re-organized new SSTables are written back into SSDs. For example, two new SSTables C and D are written into Level *i* in Figure 2.

*Step* ➄*: Old SSTable deletion.* The old SSTables are deleted when the new SSTable writes are finished. The two original SSTables in Figure 2 are deleted or marked as invalid.

Note that the all reserved KV items in this example, which are marked as yellow in Figure 2, are read into memory and then written back to the SSD, remaining unchanged. That is, they are all duplicate data viewed from the SSD level. The only change is that the data blocks in which they are stored (left vs. right figures in SSD level in Figure 2) are now different. Motivated by the remapping-based SSDs that exploit duplicate blocks, this paper considers applying the remapping approach to eliminate duplicate data movement involved in the compaction process of LSM-tree-based KV stores.

## 3. Motivation

Noticing that the size of a data block (often 4 KB) is the same as the flash page size, we start by setting the data block granularity of remapping to the flash page size. In this section, we first present the concept of unchanged data block (UDB), which refers to the data blocks that are duplicated during compaction. A preliminary experiment is performed to obtain the ratio of UDB in the real-world benchmarks.

### 3.1. Unchanged Data Block (UDB)

**Figure 3:**
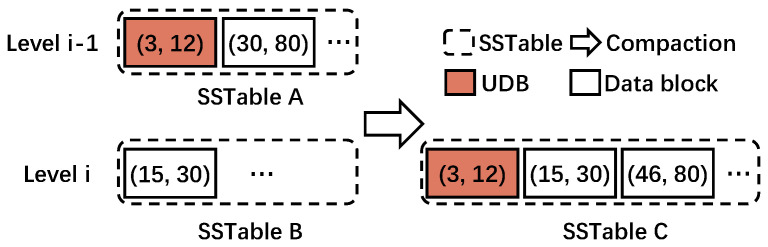
Example of UDB. ($K_{s}$, $K_{e}$) represents the key range of the KV items.

As compaction occurs in LSM-tree-based KV stores (hereafter, referred to simply as compaction), there exists data blocks whose entire KV items do not change before and after the compaction. We define such a data block as an unchanged data block (UDB). Consider the example given in Figure 3, where the red box is a UDB. In this example, we use ($K_{s}$, $K_{e}$) to represent the range of each data block in the SSTable where $K_{s}$ and $K_{e}$ are the start and end keys, respectively. Assume SSTable A in Level i−1 is chosen as the victim. As the key range of SSTable A overlaps with SSTable B in Level *i*, these two SSTables are both read into the memory and merged to form a new SSTable C, which is written back to Level *i*. Specifically, the data block in SSTable B with the key range (15, 30) overlaps with the data block in SSTable A with the key range (30, 80). Thus, they are sorted and merged in memory, into two data blocks with key ranges (15, 30) and (46, 80). It should be noted that the data block with key range (15, 30) has one outdated KV item, and thus, it is changed. As a final step, these two data blocks along with the data block with key range (3, 12), which happens to be unchanged during this entire process, are taken together to form SSTable C. We refer to this unchanged data block as a UDB.

### 3.2. Prevalence of UDB

**Figure 4:**
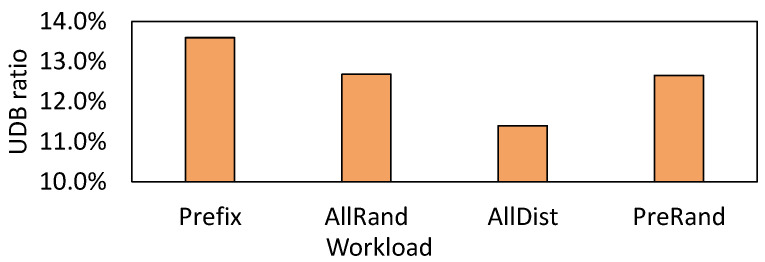
UDB ratio for real-world benchmarks.

Due to their unchanged nature, UDBs may be exploited with remapping techniques [26,27,28,29]. However, a question arises as to whether UDBs are prevalent in the real world. To answer this question, we perform a preliminary study on the Mixgraph benchmark [19]. Mixgraph is collected from Facebook’s social graph workload with query composition and key access patterns, and contains four workloads of Prefix Dist, All Random, All Dist, and Prefix Random. The details of these workloads are introduced by Cao et al. [19]. We collect the ratio of UDB by directly comparing the data blocks before and after compaction. A total of 50 million mixed read–write requests are issued in the four workloads. The results are presented in Figure 4, where we observe that the UDB ratios for the four workloads are 13.6%, 12.7%, 11.4%, and 12.7%, respectively.

These results show the prevalence of UDBs in the real world, and if we can apply the SSD remapping approach to remap these UDBs, write amplification due to compaction can be reduced, thereby improving compaction performance as well as elongating SSD lifetime. However, two challenging issues still need to be overcome to make remapping UDBs feasible. First, it is time-consuming to identify UDB by directly comparing the input SSTables with output SSTables. Thus, an efficient UDB identification method should be designed. Second, there exists semantic isolation between the application and SSD. That is, there are no explicit duplicate data write operations that expose the data movement during the compaction process. Therefore, the existing remapping interface cannot be directly used.

This paper proposes RemapCom, a remapping-based compaction method for LSM-tree-based KV stores to solve the above two issues. For the first issue, RemapCom designs a lightweight state machine integrated into the merge process of compaction to identify the UDBs. For the second issue, RemapCom provides two primitives for the application to implicitly implement the remapping operation.

## 4. RemapCom: Remapped Compaction

In this section, we present the details of RemapCom. The architectural overview is first illustrated, and then each component of RemapCom is presented in detail. Finally, the implementation of RemapCom and its overhead analysis are presented.

### 4.1. Overview of RemapCom

**Figure 5:**
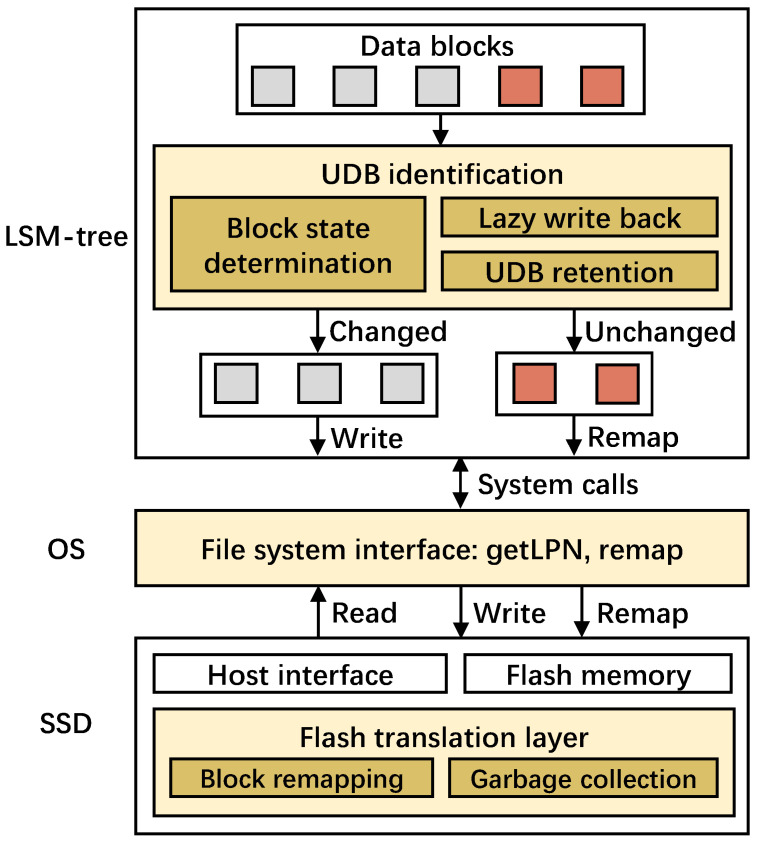
RemapCom architectural overview. Yellow boxes are the new RemapCom components added to a typical LSM-tree-based KV store execution stack.

Figure 5 presents the architectural overview of RemapCom as integrated with an existing remapping-based SSD. RemapCom comprises three main components, namely, UDB identification, implementation interfaces, and data remapping. UDB identification is designed within the application layer; that is, the LSM-tree-based KV store, with three key mechanisms— specifically, block state determination, which checks the KV items with a lightweight state machine to determine whether the data block is unchanged or not, lazy write back, which is used to buffer the KV items temporarily before the final state is determined, and UDB retention, which aims to generate more UDBs than are obvious. Implementation interfaces are designed in the file system layer with two primitives of getLPN and remap. Data remapping is implemented within the remapping-based SSD, mainly maintaining the original remapping method but considering the new primitives of RemapCom. We discuss each of the three components in the following sections.

### 4.2. Block State Determination

It is time-consuming to identify UDB by comparing directly input SSTables with output SSTables after sorting and merging. To determine the state of data block and reduce the cost of identifying the state, RemapCom utilizes a lightweight state machine which is integrated into the KV merging process of SSTable compaction. When the compaction begins, a state machine is created in memory, and it is destroyed after the last KV item is merged. The state machine determines the data block’s state as RemapCom traverses the sorted KV items during merging as described below.

**Figure 6:**
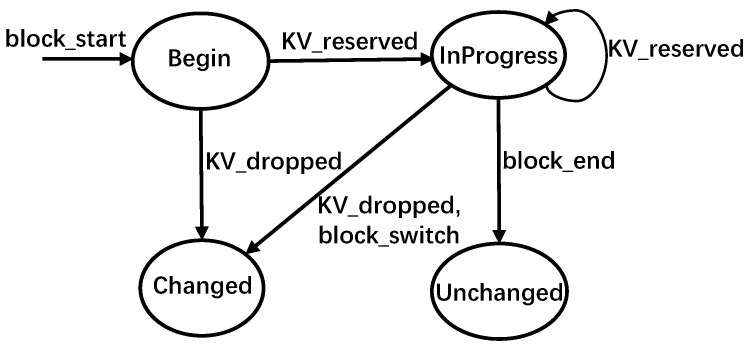
Lightweight state machine.

A data block can be in four states: *Begin*, *InProgress*, *Changed*, and *Unchanged*, as shown in Figure 6.

- *Begin*: A data block is in this state when RemapCom traverses the first KV item of the data block.
- *InProgress*: A data block is in this state when no KV changes are detected in the data block up to the current time. That is, this data block still has the potential to be a UDB.
- *Changed/Unchanged*: A data block is in either of these states once its final state has been determined by the state machine. If the state is *Unchanged*, it is a UDB. Otherwise, it is a block whose content has changed.

**Figure 7:**
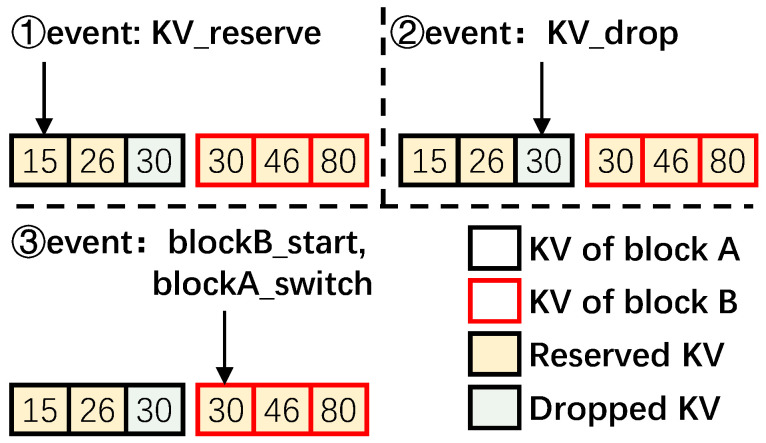
Examples of events in the state machine.

Traversing each KV item would trigger specific events in the state machine, which takes the data block into one of the above four states. These events are listed in Table 1 while the state transition diagram is given in Figure 6. We now discuss the state transitions occurring with the events using the example given in Figure 7 as necessary.

- **block_start**: This event is triggered when RemapCom traverses the first KV item of a block. Accordingly, the state of the block goes to the *Begin* state in Figure 6.
- **KV_reserved**: This event is triggered when the KV item that RemapCom is checking in the block is to be reserved. Consequently, it is moved to the data buffer. In Figure 7, the first two KV items with Key 15 and Key 26 in block A are both reserved ones. Thus, the first item switches the state from *Begin* to *InProgress*, while the second item switches from *InProgress* to *InProgress* in Figure 6.
- **KV_droppped**: This event is triggered when the KV item that RemapCom is checking in the block is to be dropped. For example, the KV item with Key 30 of block A in Figure 7 is a dropped item and to be removed during compaction. Accordingly, the state of the data block switches to *Changed*. This can happen from *Begin* if the first KV item is a dropped item, or from *InProgress*, otherwise. This means that the data block cannot be a UDB since the *Changed* state cannot switch to any other state.
- **block_switch**: This event is triggered when the RemapCom traversal moves from one block to another block. Recall that RemapCom is in the process of merging two SSTables, and this is happening with KV items in two data blocks of SSTables. From a single data block point of view, traversal by RemapCom to a different data block, that is, switching the traversal to a different block, means that the next KV item to be traversed is in a different block. This, in turn, means that the content of the newly generated block will contain KV items from two different blocks. Thus, the state of the block transitions to *Changed*. For example, a block_switch happens in Figure 6 where RemapCom traverses to Key 30 in block B after traversing Key 30 in block A.
- **block_end**: This event is triggered when the last KV item in the data block is traversed. This means that all the KV items in this block have been traversed from start to end and will be written to a new block without any change. Thus, in Figure 6, a transition from *InProgress* to *Unchanged* occurs, and the data block reaching this *Unchanged* state is a UDB.

**Table 1:** Description of events in state machine.

Event	Description
block_start	First KV item is traversed
KV_reserved	KV item being traversed now is reserved data
KV_dropped	KV item being traversed now is dropped data
block_switch	Traversal is directed to KV item in another block
block_end	Last KV item in block has been traversed

### 4.3. Lazy Write Back

A new SSTable formed through compaction is written back to the SSD. Any number of blocks in the SSTables being merged may be in *InProgress* state; that is, their final states are undecided. For RemapCom, to take advantage of remapping, write back of blocks must be delayed until their states are determined. Thus, a data buffer is used to cache the KV items of the data block when its state is *InProgress*.

The KV items are, thus, lazily written back to SSDs under two scenarios. First, when the state switches to *Changed*, the traversed KV items buffered in the data buffer are written into the output SSTable just like the original compaction process. Second, the state switches to *Unchanged*. This means the traversed KV items buffered in the data buffer form a UDB. Thus, RemapCom writes this UDB by just remapping it with SSD remapping command, instead of using the write command, to write these KV items into the output SSTable. The data buffer would be flushed into SSDs to combine output SSTable as fixed data blocks.

For the example in Figure 7, when the block stays in the judging state, the KV items are temporally buffered, and they are flushed when the final state of the current data block is determined.

### 4.4. UDB Retention

**Figure 8:**
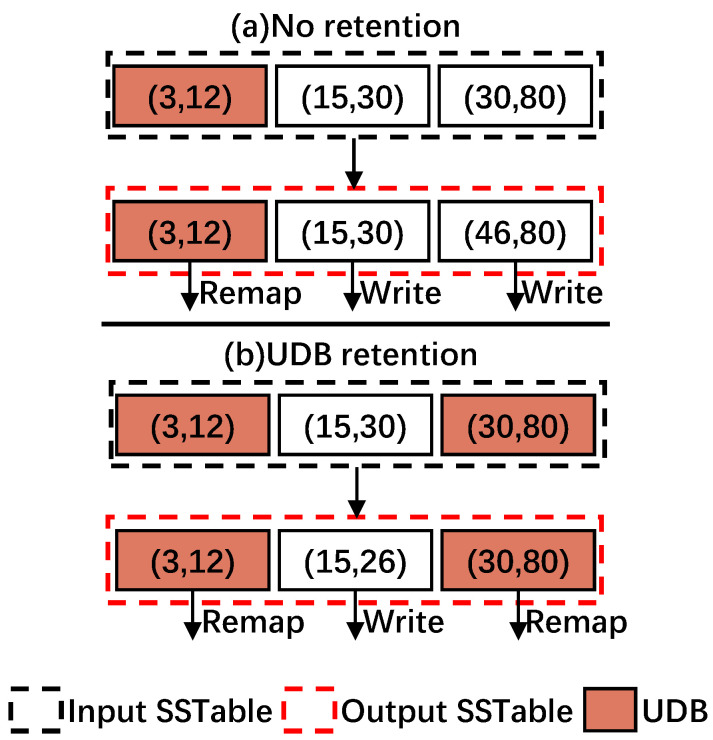
Example of UDB retention in RemapCom. The colored UDB is maintained from being split by the adjacent block.

Even if a data block is determined to be in an *Unchanged* state by the state machine, it may be split into adjacent data blocks due to the flash page alignment, eventually converting it to a changed block. For example, in Figure 8a, data blocks with key ranges of (3, 12), (15, 30), and (30, 80) are compacted into three data blocks with the key ranges of (3, 12), (15, 30), and (46, 80). Although the data block with key range (30, 80) is determined to be a UDB, one of its KV item with Key 30 is used to fill up the left space of its adjacent block.

To address this issue, RemapCom uses a strategy that we call UDB retention, which allows the UDBs determined by the state machine to remain UDBs as they are written to the SSD.

Specifically, during a compaction job, assuming there are two adjacent data blocks overlapping with each other at the key boundary ($K_{s}$ or $K_{e}$ are the same), and one of the them has no changed KV items except for the overlapping boundary that may change due to compaction, the other contains some changed KV items. Therefore, in this time, RemapCom keeps the boundary key of the former to form a UDB.

For example, in Figure 8b, UDB retention allows the block with key range (30, 80) to be retained as a UDB. With this strategy, RemapCom can generate more UDBs. The obvious reduction in write amplification also can be observed. Compared with the conventional compaction process that needs to write three data blocks, RemapCom just needs to write one data block.

However, internal fragmentation will incur in the adjacent block, wasting some storage space if the UDB retention policy is adopted. For example, as shown in Figure 8b, the original (15, 30) block will not fill entirely, being written as a (15, 26) block. That is, the total size of KV items within a data block may be smaller than the data block size. This is a trade-off between waste due to internal fragmentation and benefits by block remapping, and the final results are shown in Section 5.

### 4.5. Primitives and Data Block Remapping

Two primitives, getLPN and remap, are designed in RemapCom to exploit the remap function. getLPN obtains the logical page number (LPN) of UDB in SSDs and remap remaps the new logical page number to the old physical page number of UDB in the Flash Translation Layer (FTL), eliminating the actual write to flash memory. These two primitives are encapsulated into the file system interface, enabling the application layer to directly utilize the remap function. The detailed explanation of these two primitives are listed as follows:

- getLPN(fileno,offset): This primitive obtains the start LPN of the UDB. Given the SSTable’s file number, fileno, and the UDB’s offset in SSTable, offset, the LPN of the UDB in SSD is obtained by using the *ioctl* system call.
- remap(src_LPN,dst_LPN,length): This primitive sends the remap signal to the SSD along with the necessary information for remapping: src_LPN, representing the original start LPN of the UDB, dst_LPN, representing the new start LPN of the UDB, and length, representing the number of pages involved in the UDB. Thus, the UDB with LPNs between src_LPN and src_LPN + length − 1 is remapped to the area between dst_LPN and dst_LPN + length − 1. There are no extra write operations, and it only involves changes about several mapping entries.

To support these two primitives, we extend the nvme_ioctl system call.

With these two primitives, there are three main steps for remapping the UDBs in SSDs, as illustrated below.

1. Obtain the logical page number corresponding to the UDB in SSD by calling getLPN.
2. Send remap requests to the file system by calling remap. For the example in Figure 8a, RemapCom uses remap command to notify SSD that the UDB with the key range (3, 12) needs to be remapped.
3. Handle remap requests in SSDs. With the assistance of the remap primitive, the SSD controller can easily perform the remapping just like Remap-SSD [28].

### 4.6. Overhead Analysis

The overhead of our proposed RemapCom method is analyzed from three aspects.

**State machine:** RemapCom uses less than 4 bytes of memory for a state machine. Since KV items in an SSTable have no overlapping keys, only one state machine is needed for each SSTable, and the memory space of state machine is freed after compaction. Therefore, the maximum number of state machines in memory is the number of input SSTables in the compaction. As a result, this overhead is minimal.

**Lazy write back:** The lazy write back strategy requires data buffer in memory to store the temporary KVs from the block while in the InProgress state. Its size is less than one data block. The extra storage space of RemapCom can be ignored.

**Remapping-based SSDs:** According to existing studies, the remapping-based SSD needs a remap table to ensure mapping consistency [26,27,28,30]. In brief, the remap table is stored in byte-addressable NVRAM. The SSD allocates an NVRAM segment for the blocks that contain remapped pages. A new segment is not allocated until the previous segment of the block is filled with remap entries. The remap entry has two fields: flash page offset in block and remap LPN. It only takes 6 bytes to represent a remap entry for 256 GB SSD (16 bits for the first field and 32 bits for the second field) and the entry can be extended to 8 bytes if the SSD is larger. Segments can be cleared and reused after the block to which the segment belongs is garbage-collected. So the overhead of remapping one page of SSD is appending an extra remap entry to the remap table. It is worthwhile to use NVRAM to exploit the SSD remap strategy because remapping 4 KB flash page only produces 6 B NVRAM, which is about 1.5% the storing cost of SSD. RemapCom utilizes the same remapping method as these studies, so the overhead of the remap table is acceptable.

## 5. Evaluation

This section evaluates the proposed RemapCom method. The experimental setup is first illustrated and results on microbenchmarks are then shown and analyzed. Finally, the evaluation results on real-world benchmarks are illustrated and analyzed.

### 5.1. Experimental Setup

Our experiments are conducted on FEMU [32], a popular NVMe SSD emulator based on QEMU. The configurations of our experiments are summarized in Table 2. The machine running FEMU is equipped with Intel(R) Xeon(R) Gold 6226R CPU @2.90 GHz and 128 MB DRAM. The Linux kernel of FEMU host system is Linux 5.15 and we format the emulated SSD as an EXT4 file system.

The emulated SSD has a capacity of 16 GB. The flash page size is 4 KB and latencies of page read, page write, and block erase are 40 μs, 200 μs, and 2 ms, respectively.

For the LSM-tree-based KV store, we make use of version 1.23 of LevelDB. The parameters of LevelDB are set as follows. The block size is set to 4 KB, and the SSTable size is set to 2 MB. We denote the original LevelDB as “Baseline”, LevelDB only with block state determination and UDB remapping, but no UDB retention as “RemapCom-Base”, and LevelDB, with all components of RemapCom as “RemapCom”.

### 5.2. Results on Microbenchmarks

In this section, we use the db_bench microbenchmark for evaluation. We measure the UDB ratio, write amplification, and write performance of RemapCom by randomly writing KV items with two different distributions to the database. Since the *fillrandom* workload of db_bench can only generate random key–value items, we add *zipfian* distribution, which produces key–value pairs that conform to the principle of locality.

The total size of the KV items written by the host is 4 GB while the number of KV items written varies depending on the value size. For example, if the size of KV items is 4 KB, then one million KV items will be written. The key size is fixed to 16 bytes while the value size varies according to the KV item size, which ranges from 256 B to 4 KB.

**Figure 9:**
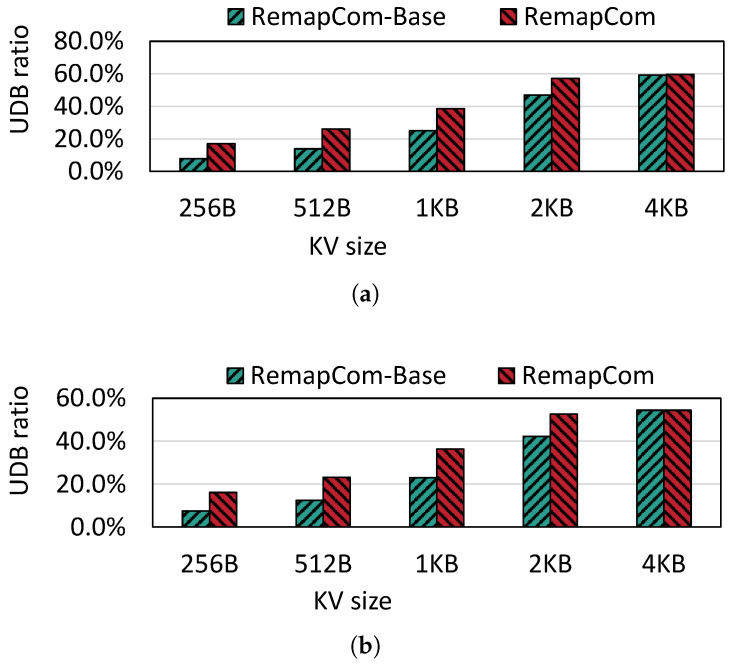
UDB ratio for different KV sizes. (**a**) Fillrandom. (**b**) Zipfian.

**UDB Ratio:** Figure 9 shows the UDB ratio during compaction for the random write workload from db_bench over various KV item sizes varying from 256 B to 4 KB. We observe that when the KV size exceeds 1 KB, the ratio is higher than 25% for RemapCom-Base. As the KV size increases, the ratio of UDB in SSTable which is participating in compaction increases. This is because the number of KV items in a fixed 4 MB block size becomes smaller as the KV size increases. From the state machine in Section 4.2, it can be understood that the state of the data block is determined to be *Changed* as long as any changed (i.e., invalid) KV item is found while traversing a data block. However, the *Unchanged* state requires that all KV items within this data block remain unchanged. The probability of each KV item being invalid is the same. Therefore, a larger number of KV items increases the higher probability that the data block will be in the *Changed* state. So, the UDB ratio will increase as the number of KV items decreases due to the larger KV size. We also observe that the UDB ratios of RemapCom for KV sizes 256 B, 512 B, 1 KB, and 2 KB are 1.11×, 1.15×, 1.18×, and 1.33× higher than that of RemapCom-Base, respectively. This is because the UDB retention strategy prevents UDB from mixing with adjacent data blocks. When the KV size equals 4 KB, the results of UDB retention are not obvious, as there is hardly any block mixture because the KV size is equivalent to the block size.

**Figure 10:**
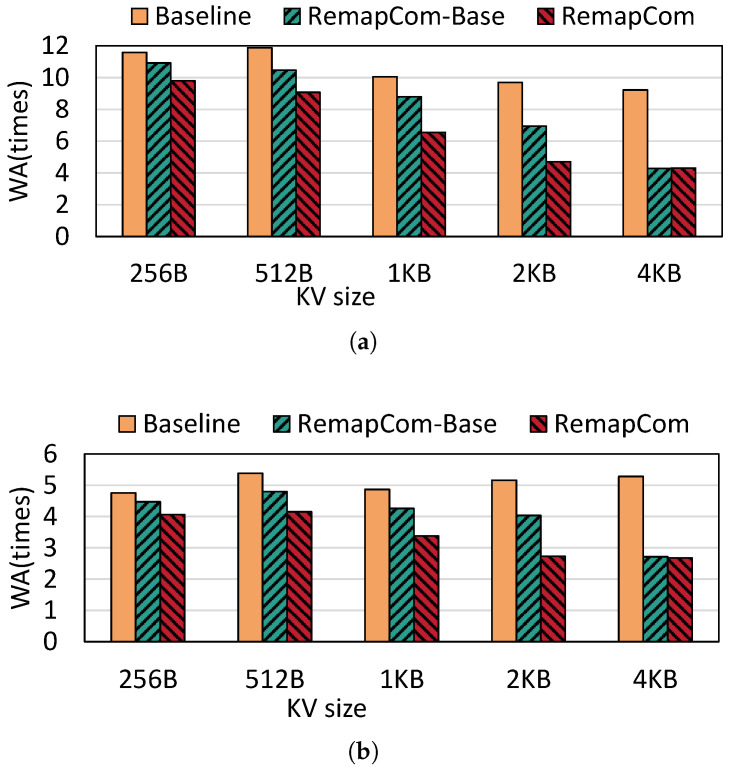
Write amplification for different KV sizes. (**a**) Fillrandom. (**b**) Zipfian.

**Write amplification:** Figure 10 shows the write amplification of LevelDB with random writes under different KV sizes. Compared to *fillrandom*, random writes under *zipfian* distribution show 2.06× lower write amplification than *fillrandom* on average. This is because KV items written by the zipfan distribution have access locality, and those hot KV items are more likely to stay at the higher level, thus reducing write amplification. Compared with LevelDB, RemapCom-Base and RemapCom both reduce write amplification significantly due to the remapping of the UDB. RemapCom-Base reduces SSD writes by 22.4% in *fillrandom* distribution and 20% in *zipfian* distribution, on average. RemapCom eliminates SSD writes of these two types of benchmarks by 35.7% and 32.8% on average, respectively. As the KV size increases, the benefit of RemapCom-Base and RemapCom in write amplification becomes more pronounced. This is because there is a larger ratio of UDB for larger KV size, as shown in Figure 9, and it can be observed that RemapCom-Base and RemapCom show nearly the same WA at the KV size of 4 KB. This is because the size of data block in LevelDB is set to 4 MB by default, and there is only one 4 KB-sized KV item in every data block. Therefore, the effect of UDB retention is limited, which results in minor differences between RemapCom-Base and RemapCom.

We also conduct a detailed analysis of the write amplification in each level of LevelDB. Table 3 shows the SSD writes reduced by RemapCom-Base and RemapCom compared to LevelDB in each level for 1 MB KV size. We find a higher reduction in write amplification for higher levels. This is because data blocks in higher levels are colder. This means that as the level goes up, it becomes increasingly possible that the UDBs from the lower level trickle up to the upper level, having an accumulation effect of UDBs. Thus, more UDBs achieve more reduction in write amplification.

**Figure 11:**
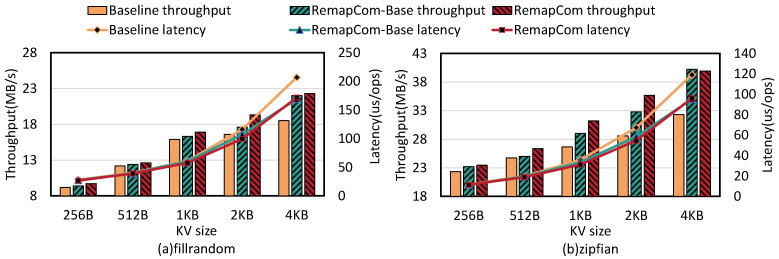
Write performance on db_bench with various KV sizes.

**Write performance:** Figure 11 shows the random write throughput and latency of LevelDB, RemapCom-Base, and RemapCom for various KV sizes. Compared to LevelDB, RemapCom-Base improves write throughput by up to 30% with *fillrandom* and 25% with zipfian distribution. The reason is that the write performance of LevelDB is strongly affected by the background compaction process of writing SSTables. RemapCom avoids writing UDBs in SSTables, thus mitigating the write performance degradation caused by compaction. However, the improvement by RemapCom-Base compared to the baseline is only 2.1∼6% when the KV size is less than 4 MB. With RemapCom, which incorporates UDB retention, this improvement reaches 5.4∼16.2%. The improvement in write performance by RemapCom becomes more pronounced as the KV size increases. This is because when the KV size increases, the effect of RemapCom on write amplification reduction becomes more significant. It can be observed that the baseline latency increases more. This is because of the UDB retention policy. As mentioned in UDB ratio changes, for the 4 MB-sized data block, the larger KV leads to fewer KVs in every data block. Therefore, it is not easier to be in the Changed state along with a higher UDB ratio, as the KV size is larger. Moreover, the benefit of UDB retention policy becomes more obvious compared to the baseline, so the baseline latency increases more.

### 5.3. Real-World Benchmarks

In this section, we consider the performance of RemapCom on two real-world workloads; namely, the YCSB [18] and Mixgraph [19] benchmarks.

The YCSB benchmark is a widely used macrobenchmark suite delivered by Yahoo!. The characteristics of the YCSB benchmark workload are given in Table 4. For these experiments, we first load 800 MB of KV items into the KV store. Then, we run the six workloads, A through F, provided by YCSB on the KV store. In the experiments, we consider three different sizes of KV items: 256 MB, 1 MB and 4 MB, which represent small, middle, and large KV items, respectively.

**Figure 12:**
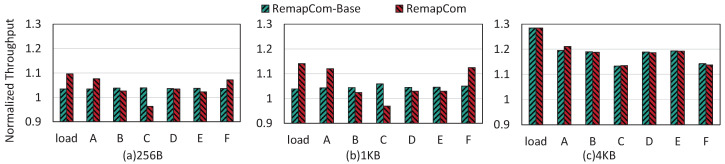
Throughput of RemapCom normalized to LevelDB for YCSB.

Figure 12 shows the throughput results of RemapCom-Base and RemapCom normalized to the baseline, where we can obtain the following observations.

1. For the Load workload, RemapCom-Base increases the throughput by 3.4%, 3.9%, and 28% compared to LevelDB for the three KV sizes, respectively. For RemapCom, the improvements are 9.7%, 14.1%, and 28%, respectively.
2. For the write-intensive workloads A and F, compared to LevelDB, RemapCom’s improvements are 7.6%, 12%, and 21.1% for workload A and 7.1%, 12.4%, and 13.8% for workload F, for the respective KV sizes.
3. For the read-intensive workload C, we find that read performance in RemapCom-Base obtains 3.9%, 5.9%, and 13.3% improvement for the three KV sizes, respectively. The reason is that the read operations can trigger seek compaction [7], where UDB can also be remapped. However, compared to LevelDB, RemapCom reduces throughput by 3.8% and 3.1% for 256 MB and 1 MB KV sizes, respectively. This is because UDB retention separates UDB from adjacent blocks, which leads to a certain amount of read and write amplification. As mentioned in Section 4.4, the total size of KV items within a data block may be smaller than the size of data block, i.e., internal fragmentation resulted from UDB retention policy causes a certain degree of space waste, which reduces the read performance.
4. RemapCom also achieves an improvement in throughput for workloads with only a small portion of writes (i.e., B, D, and E). The average improvements of all KV sizes are 2.5%, 3.2%, and 2.6% for the B, D, and E workloads, respectively. These results show that RemapCom can also improve overall performance for read-intensive workloads.

**Figure 13:**
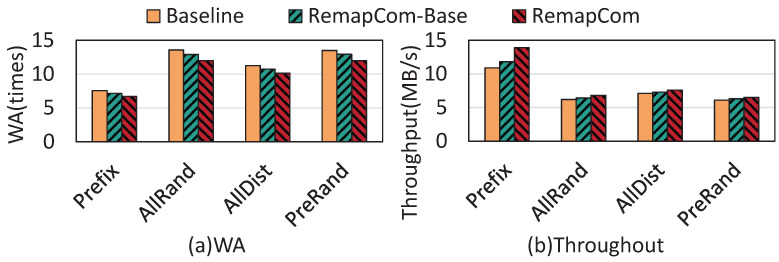
Results of Mixgraph benchmark.

The other real-world workload is Mixgraph, a social-graph benchmark from Facebook that contains four workloads: Prefix, AllRand, AllDist, and PreRand [19]. In these experiments, all four workloads issue 50 million query requests. The test results are shown in Figure 13.

From the figure, we observe that compared to LevelDB, RemapCom-Base and RemapCom reduce write amplification by 4.5% and 11%, on average. As the benchmark has a large proportion of small KV items, the benefit of RemapCom is not significant. In terms of throughput, compared with the baseline, RemapCom-Base is 1.08×, 1.03×, 1.03×, 1.03× faster for the four workloads, respectively, while the improvement of RemapCom is 1.28×, 1.10×, 1.07×, 1.07×, respectively.

The above results indicate that RemapCom shows higher write performance than existing works in real-world benchmarks.

### 5.4. Sensitivity Study

In this section, we analyze two factors that affect RemapCom, which are the write size and the scale of KV items.

**Figure 14:**
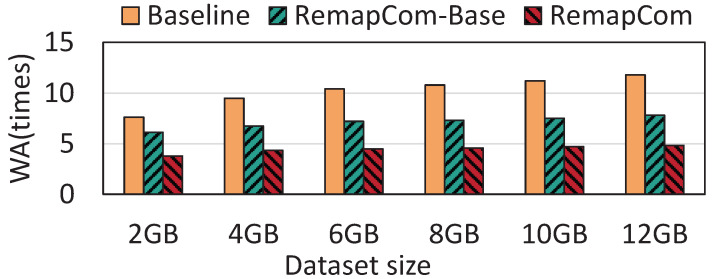
Results with different dataset sizes.

**The size of the dataset:** We use the *fillrandom* workload of db_bench to evaluate the performance under the different sizes of datasets. The size of KV item is fixed to 4 KB while the number of KV items written varies depending on the size of dataset. We start with 2 GB KV items and gradually increase to 12 GB, testing performance under different dataset sizes separately (i.e., 2 GB, 4 GB, 6 GB, 8 GB, 10 GB, and 12 GB). Figure 14 shows the WA results of six datasets with different sizes. The results show that as the size of the write dataset increases, the write amplification becomes more serious for LevelDB while RemapCom-Base and RemapCom are slightly affected.

**Figure 15:**
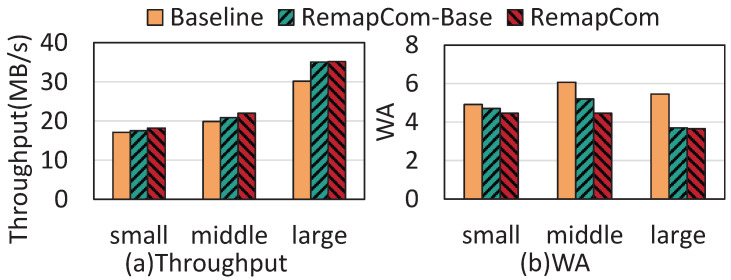
Results under varying KV sizes.

**Varying the size of KV items:** In the real world, the size of the KV item is supposed to be variable [19]. To study the effectiveness of RemapCom in different KV size scales, we modify the db_bench to generate KV items with varying sizes within three ranges of 100 B–1 KB, 1 KB–4 KB, and 4 KB–16 KB, which represent small-, middle-, and large-scale KV sizes, respectively. Figure 15 shows the results of random write throughput and write amplification for workloads with small, middle, and large KV sizes. The write throughput of RemapCom is 1.06×, 1.11×, and 1.17× higher than LevelDB for the three sizes, respectively, and the write amplification is 1.1×, 1.36×, and 1.49× lower than LevelDB for the three sizes, respectively. This indicates that RemapCom can achieve better performance than the baseline under large KV sizes.

## 6. Related Work

We categorize three aspects of related works in optimizing the performance of LSM-trees.

**(1) Adding NVMs to LSM-trees in DRAM-SSD storage system.** SplitDB utilizes a fast NVM to store frequently accessed, small-sized high level data [33]. MioDB replaces the on-disk data structure of LSM-trees with persistent skiplists so that the operations of flush and compaction can be finished by using an efficient *memcopy()* [34]. MatrixKV places the data in Level 0 into NVM by designing a structure of the matrix container [15]. SLM-DB uses only one level to store SSTables and maintains the key address in NVM with B+ tree [35]. These approaches effectively reduce write amplification and improve the write performance of LSM-tree-based KV stores.

**(2) Optimizing compaction performance of LSM-trees.** In order to reduce the effect of frequently updated small KV items during compaction, L2SM removes the hotter and sparser KV items at an early stage [11]. Sun et al. propose to avert rewriting data that do not need to be updated during compaction into SSDs [36]. Chai el al. propose a novel Lower-level Driven Compaction (LDC) method which breaks the limitations of the traditional upper-level driven compaction manner and triggers practical compaction actions bottom-up [37]. Shetty et al. propose a novel workload-independent data structure called the VT-tree which extends the LSM-tree to efficiently handle sequential and file-system workloads [38], but the software to apply remapping leads to data error when the power is cut off. Dayan et al. introduce a novel compaction granularity method, Spooky, to merge a set of fully overlapping files at once, limiting space amplification and compaction overhead [39]. Thonagi et al. propose a new algorithm to decide whether to perform a partial merge to mitigate the compaction performance [40]. Hu et al. propose to limit the SSTables that participate in compaction, thus mitigating the compaction performance [41]. Lee et al. propose a new Compaction-Aware Zone Allocation algorithm (CAZA) that allows the newly created SSTables to be deleted together after merging in the future [42]. Wang et al. propose a novel compaction scheme named Block Compaction that adopts a block-grained merging policy to perform compaction operations [43]. Jung et al. propose a new Lifetime-Leveling Compaction (LLC) to avoid the newly generated SSTables from being selected for compaction soon, which reduces the number of short-lived SSTables [44]. Wu et al. propose to consider in advance the write amplification that SSTable may cause during GC while selecting the victim SSTable for compaction [45]. As most of these mechanisms do not consider the compaction algorithm of LSM-tree by using data remapping strategy, RemapCom can be integrated with these approaches.

**(3) Redesigning the structure of LSM-trees for SSDs.** KVSSD upgrades the existing logical-to-physical (L2P) mapping of the FTL to key-to-physical (K2P) mapping and implements no-copy SSTable compaction through remapping of KV items [14,46,47]. As the FTL is changed, KVSSD can only be used specifically for LSM trees and cannot be applied to other applications. By contrast, RemapCom does not specialize FTL for LSM-tree by just adding a new system call. Therefore, it can be applied to all applications. PebblesDB presents a novel data structure called Fragmented Log-Structured Merge Trees that allows appending to the SSTables instead of merging them to the next level [48]. WiscKey separates keys and values so that it can reduce the overhead of rewriting values of KV items [6,49]. However, it needs a special garbage collector to reclaim free space in the value log. Fan et al. propose a new remap strategy which uses the characteristics of compaction in LSM-tree to reduce the write back overhead of unchanged data [50].

## 7. Conclusions

In this paper, in order to improve compaction performance of LSM-tree-based KV stores, we propose an LSM-tree-based KV store for SSDs, named RemapCom, to reduce write amplification and improve performance by leveraging data block remapping during compaction. RemapCom identifies unchanged data blocks (UDB) of SSTables through a lightweight state machine, increases the ratio of UDB to fully exploit the benefits of remapping, and designs two primitives to implement block remapping. The results on comprehensive benchmarks have verified its effectiveness in reducing write amplification and optimizing write performance.

## Figures and Tables

**Table 2:** Experiment setup.

Host Configuration	
CPU	Intel(R) Xeon(R) Gold 6226R CPU@2.90 GHz
Memory	128 MB DRAM
OS	Ubuntu 20.04 (kernel version 5.15.0)
**Guest Configuration**	
CPU	4vCPU
Memory	16 MB DRAM
OS	Ubuntu 20.04 (kernel version 5.15.0)
File system	EXT4
NVMe SSD	16 MB
**Flash Memory Configuration**	
Page size	4 KB
Page read latency	40 μs
Page program latency	200 μs
Page erase latency	2000 μs

**Table 3:** Write amplification reduction in different levels.

Level	1	2	3	4
**RemapCom-Base**	1%	26%	29%	32%
**RemapCom**	5%	47%	52%	53%

**Table 4:** Description of YCSB workload.

Workload	Operations and Distribution
Load	100% inserts, uniform
A	50% updates, 50% reads, zipfian
B	5% updates, 95% reads, zipfian
C	100% reads, zipfian
D	5% inserts, 95% reads, latest
E	5% updates, 95% scans, zipfian
F	50% reads, 50% read-modify-writes, zipfian

## Data Availability

The data presented in this study are available on request from the corresponding author. The data are not publicly available due to confidentiality request.
